# Supplementary material for: A novel genetic strategy to interrogate an unknown phenotypic modifier: Sdhc KO-Robertsonian mice develop frequent thyroid abnormalities with papillary thyroid carcinoma-like features
Source: PLoS One. 2026 Mar 31;21(3):e0336731. doi: 10.1371/journal.pone.0336731 (PMC13038002; doi:10.1371/journal.pone.0336731)
Supplement: Attachment — Submitted filename: Response to Reviewers.docx [file pone.0336731.s002.docx]

PLOS One PONE-D-25-58546

A novel genetic strategy to interrogate an unknown phenotypic modifier: an Sdhc KO-Robertsonian mouse with a semi-homologous chromosome develops papillary thyroid carcinoma-like tumours

PLOS One

Journal Requirements:

1. When submitting your revision, we need you to address these additional requirements.

2. To comply with PLOS ONE submissions requirements, in your Methods section, please provide additional information regarding the experiments involving animals and ensure you have included details on (a) methods of sacrifice, and (b) efforts to alleviate suffering.

Reply: Further details have now been added (lines 147-150)

3. We note that the grant information you provided in the ‘Funding Information’ and ‘Financial Disclosure’ sections do not match.

When you resubmit, please ensure that you provide the correct grant numbers for the awards you received for your study in the ‘Funding Information’ section.

Reply: This has now been corrected.

4. Thank you for stating the following financial disclosure:

Dutch Cancer Foundation (KWF UL 2011-5025) and The Paradifference Foundation

Please state what role the funders took in the study. If the funders had no role, please state: "The funders had no role in study design, data collection and analysis, decision to publish, or preparation of the manuscript."

If this statement is not correct you must amend it as needed.

Please include this amended Role of Funder statement in your cover letter; we will change the online submission form on your behalf.

Reply: This has now been corrected in both the manuscript and the cover letter.

5. We note that your Data Availability Statement is currently as follows:

All relevant data are within the manuscript and its Supporting Information files.

Reply: All previously missing data and methods have now been added, or references to missing data deleted.

6. We note that you have included the phrase “data not shown” in your manuscript.

Reply: References to this data have now been removed from the manuscript.

7. Please amend either the abstract on the online submission form (via Edit Submission) or the abstract in the manuscript so that they are identical.

Reply: Now amended.

8. Your ethics statement should only appear in the Methods section of your manuscript. If your ethics statement is written in any section besides the Methods, please move it to the Methods section and delete it from any other section. Please ensure that your ethics statement is included in your manuscript, as the ethics statement entered into the online submission form will not be published alongside your manuscript.

Reply: Now amended (line 115).

9. Please upload a new copy of Figures 1 and 2, as the detail is not clear. Please follow the link for more information: https://journals.plos.org/plosone/s/figures

10. If the reviewer comments include a recommendation to cite specific previously published works, please review and evaluate these publications to determine whether they are relevant and should be cited. There is no requirement to cite these works unless the editor has indicated otherwise.

Additional Editor Comments:

Address all the comments carefully, particularly Reviewer 1. Any unaddressed comments will result in re-revision.

Reviewer's Responses to Questions

Comments to the Author

1. Is the manuscript technically sound, and do the data support the conclusions?

The manuscript must describe a technically sound piece of scientific research with data that supports the conclusions. Experiments must have been conducted rigorously, with appropriate controls, replication, and sample sizes. The conclusions must be drawn appropriately based on the data presented.

Reviewer #1: Yes

Reviewer #2: Yes

________________________________________

2. Has the statistical analysis been performed appropriately and rigorously?

Reviewer #1: Yes

Reviewer #2: Yes

________________________________________

3. Have the authors made all data underlying the findings in their manuscript fully available?

The PLOS Data policy requires authors to make all data underlying the findings described in their manuscript fully available without restriction, with rare exception (please refer to the Data Availability Statement in the manuscript PDF file). The data should be provided as part of the manuscript or its supporting information, or deposited to a public repository. For example, in addition to summary statistics, the data points behind means, medians and variance measures should be available. If there are restrictions on publicly sharing data—e.g. participant privacy or use of data from a third party—those must be specified.

Reviewer #1: Yes

Reviewer #2: Yes

________________________________________

4. Is the manuscript presented in an intelligible fashion and written in standard English?

PLOS ONE does not copyedit accepted manuscripts, so the language in submitted articles must be clear, correct, and unambiguous. Any typographical or grammatical errors should be corrected at revision, so please note any specific errors here.

Reviewer #1: Yes

Reviewer #2: Yes

________________________________________

5. Review Comments to the Author

Please use the space provided to explain your answers to the questions above. You may also include additional comments for the author, including concerns about dual publication, research ethics, or publication ethics. (Please upload your review as an attachment if it exceeds 20,000 characters)

**Reviewer #1**: This study presents a genetically elegant and technically demanding strategy combining an Sdhc knockout allele with a Robertsonian (Rb(1:7)) fusion chromosome to interrogate potential modifier effects and parent-of-origin mechanisms relevant to SDH-associated tumorigenesis. Although the model does not recapitulate PPGL, the emergence of thyroid lesions with PTC-like morphology is unexpected and scientifically interesting. Overall, the study is scientifically solid and methodologically sound, and it provides valuable data despite not fully achieving the intended tumor phenotype. I believe the manuscript is suitable for publication after the authors address the points below.

Specific Comments

• The central mechanistic assumption—that whole-chromosome loss contributes to the observed synergy—remains inferential. Even limited validation (FISH, CNV, allele-specific PCR) in a subset of samples would strengthen the conclusions. If additional experiments are not feasible, please explicitly acknowledge this limitation and discuss alternative explanations.

Reply: We refer to a mechanistic assumption underlying the Hensen Model, which is based on five publications that all showed loss of chromosome 11 in paragangliomas. These references [5-10] can be found on line 71. However, these findings do not appear relevant to the current data as we did not observe the expected phenotype. We used the word ‘synergy’ once in the manuscript, referring to thyroid abnormalities. This was based on differences in the frequency of thyroid abnormalities across cohorts and implied an interaction between the Rb chromosome and heterozygous loss of *Sdhc* via KO. This is not the same as chromosome loss, for which we have no evidence here. We have added the following, line 443: “We did not further explore the molecular basis of this finding, so cannot comment on whether it was associated with chromosomal loss or was a consequence of altered gene expression from metacentric Robertsonian versus telocentric chromosomes”.

• Since the study references the Hensen hypothesis and parent-of-origin effects, the manuscript should clearly state whether the Sdhc knockout allele was inherited paternally or maternally, whether reciprocal crosses were performed or considered, and how this influences interpretation.

Reply: We previously stated “These crosses ensured inheritance of Sdhc via either the female (4&6) or male (5&7) line in order to model human patterns of inheritance. The two cohorts carrying the paternally-transmitted Sdhc KO (4&6) simulate patients at risk of tumour development due to a paternally-inherited SDHD or SDHAF2 variant (Figure 1c)”. We have now made this more explicit from line 203 onwards.

• The thyroid lesions resemble papillary thyroid carcinoma, but molecular characterization is limited. Please temper the terminology by using phrases such as “PTC-like features” or “PTC-like morphology” unless molecular markers (e.g., TTF1, PAX8, BRAF) can be provided.

Reply: Indeed. We have now modified the title and text to better acknowledge this shortcoming.

• A brief and balanced discussion of why this model did not generate PPGL, and how this informs future SDH-related tumor models, would improve the clarity of the Discussion section.

Reply: The million-dollar question! We have now added a section to the discussion (Rodent models of PPGL) from line 398. Our guess: it’s the biochemistry.

• Minor issues include improving the resolution or annotation of some histology figures, ensuring consistent gene nomenclature for mouse vs. human, clarifying sample sizes in figure legends, and minor proofreading. A short comparison with previously published SDH mouse models would further help contextualize the novelty of the work.

Response:
(i) “resolution or annotation of some histology figures”; Reply: Looking through the figures they appear to us of adequate or even good resolution. We have reworked most of the figures.
(ii) “consistent gene nomenclature for mouse vs. human” ; Reply: we have now checked and corrected these inconsistencies.
(iii) “clarifying sample sizes in figure legends” ; Reply: we have now added actual mouse numbers to figures 3, 5 and 6.
(iv) “minor proofreading” ; Reply: we have carefully edited the manuscript.
(v) “short comparison with previously published SDH mouse models” ; Reply: We have now added a section to the discussion (Rodent models of PPGL) from line 398.

**Reviewer #2:** [1] How to perform immunohistochemistry? Description of immunohistochemistry is required (Figure 9g).

Reply: Apologies for this oversight. A section has now been added to Methods.

[2] Why PCR data is not available?

Reply: The gels were of poor quality and not fit for display. We have therefore removed the sentence referring to this data.

[3] It is required to show scale bars for Figure 7 to Figure 9; magnification is not enough.

Reply: This has now been corrected.

[4] Current challenge would be, an establishment of mouse model to study paraganglioma, and authors partially achieved that goal. However, there is no clear statement of perspective directions. Having this work, how authors would investigate?

Reply: The million-dollar question! We have now added a section to the discussion (Rodent models of PPGL) from line 398. Our guess: it’s the biochemistry.
